# Stopping and restarting PrEP and loss to follow‐up among PrEP‐taking men who have sex with men and transgender women at risk of HIV‐1 participating in a prospective cohort study in Kenya

**DOI:** 10.1111/hiv.13237

**Published:** 2022-01-27

**Authors:** Elizabeth Wahome, Anders Boyd, Alexander N. Thiong’o, Khamisi Mohamed, Tony Oduor, Evans Gichuru, John Mwambi, Elise van der Elst, Susan M. Graham, Maria Prins, Eduard J. Sanders

**Affiliations:** ^1^ KEMRI/Wellcome Trust Research Programme Centre for Geographic Medicine Research–Coast Kilifi Kenya; ^2^ Public Health Service of Amsterdam Department of Infectious Diseases Amsterdam The Netherlands; ^3^ Stichting HIV Monitoring Amsterdam The Netherlands; ^4^ Department of Global Health University of Amsterdam Amsterdam The Netherlands; ^5^ Departments of Global Health, Medicine, and Epidemiology University of Washington Seattle Washington USA; ^6^ Amsterdam UMC Department of Infectious Diseases Amsterdam Institute for Infection and Immunity (AII) University of Amsterdam Amsterdam The Netherlands; ^7^ Nuffield Department of Medicine University of Oxford Headington UK

**Keywords:** HIV‐1, Kenya, lost to follow‐up, MSM, predictors, PrEP

## Abstract

**Objective:**

To assess frequency and predictors of switching between being on and off PrEP and being lost to follow‐up (LTFU) among men who have sex with men (MSM) and transgender women (TGW) with access to PrEP services in Sub‐Saharan Africa.

**Methods:**

This was a prospective cohort study of MSM and TGW from coastal Kenya who initiated daily oral PrEP from June 2017 to June 2019. Participants were followed monthly for HIV‐1 testing, PrEP refill, risk assessment and risk reduction counselling. Follow‐up was censored at the last visit before 30 June 2019, or the last HIV‐1‐negative visit (for those with HIV‐1 seroconversion), whichever occurred first. We estimated transition intensities (TI) and predictors of switching: (i) between being off and on PrEP; and (ii) from either PrEP state and being LTFU (i.e. not returning to the clinic for > 90 days) using a multi‐state Markov model.

**Results:**

In all, 134 participants starting PrEP were followed for a median of 20.3 months [interquartile range (IQR): 7.7–22.1]. A total of 49 (36.6%) people stopped PrEP 73 times [TI = 0.6/person‐year (PY), 95% confidence interval (CI): 0.5–0.7] and, of these, 25 (51.0%) restarted PrEP 38 times (TI = 1.2/PY, 95% CI: 0.9–1.7). In multivariable analysis, stopping PrEP was related to anal sex ≤ 3 months, substance‐use disorder and travelling. Restarting PrEP was related to non‐Christian or non‐Muslim religion and travelling. A total of 54 participants were LTFU: on PrEP (*n* = 47, TI = 0.3/PY, 95% CI: 0.3–0.5) and off PrEP (*n* = 7, TI = 0.2/PY, 95% CI: 0.1–0.4). In multivariable analysis, becoming LTFU while on PrEP was associated with secondary education or higher, living in the area for ≤ 1 year, residence outside the immediate clinic area and alcohol‐use disorder.

**Conclusions:**

Switching between being on and off PrEP or becoming LTFU while on PrEP was frequent among individuals at risk of HIV‐1 acquisition. Alternative PrEP options (e.g. event‐driven PrEP) may need to be considered for MSM and TGW with PrEP‐taking challenges, while improved engagement with care is needed for all MSM and TGW regardless of PrEP regimen.

## INTRODUCTION

Daily oral pre‐exposure prophylaxis (PrEP) with tenofovir‐disoproxil‐fumarate and emtricitabine has been recommended for individuals at substantial risk of HIV‐1 acquisition [1]. Most countries in Sub‐Saharan Africa (SSA), including Kenya, have launched policies and guidelines on PrEP programming among priority populations, which include men who have sex with men (MSM) [2, 3]. In Kenya, PrEP is offered as part of a combination prevention package, including condoms, lubricants, risk reduction counselling and screening for symptomatic sexually transmitted infections (STIs) [1, 2]. Both maintaining PrEP use and adhering to PrEP during at‐risk behaviour are important for effective HIV‐1 prevention [4]. However, recent data suggest that continuation and adherence to daily oral PrEP are challenging in SSA, particularly for MSM and transgender women (TGW) [5, 6, 7], and could render PrEP programmes ineffective in HIV‐1 prevention.

A systematic review of 18 randomized controlled trials and implementation studies examining adherence to PrEP identified several barriers to PrEP non‐adherence among varying populations, including risk perception, side effects and stigma, social economic factors, daily life challenges and medication regimen [8]. In two recent studies among MSM, and MSM and TGW followed in coastal Kenya, 15% [9] and 6% [5] of participants, respectively, were taking sufficient daily PrEP to achieve protective drug concentrations in dried blood spot samples. While the need to remain HIV‐1‐negative and protect partners has motivated MSM to start PrEP, changes due to HIV‐1 risk perception have led to discontinuation of PrEP [10]. As a number of factors, including individual behaviour vulnerability to HIV‐1, can fluctuate over time, PrEP use patterns can also change in parallel, leading to temporary cessation and resumption of PrEP.

The objectives of the present study were to assess the frequency and determinants of switching between being on and off PrEP, as well as being lost to follow‐up (LTFU), among PrEP‐taking MSM and TGW with access to PrEP services in coastal Kenya. As switching between being on and off PrEP could be a reflection of increased or decreased sexual risk behaviour, respectively, we also aimed to compare sexual behaviours between PrEP states over time in this cohort.

## METHODS

### Study design and participants

Between June 2017 to June 2019, HIV‐1‐negative participants were being followed up in a previously well‐characterized HIV‐1 vaccine efficacy preparedness open prospective cohort study at the Kenya Medical Research Institute (KEMRI) clinic [11]. The study clinic is located in Mtwapa town, on the Mombasa/Malindi road, approximately 20 km north of Mombasa [11]. Recruitment of participants into the study was done by 10–15 trained peer mobilizers through their personal networks and at venues where male sex workers and men (or individuals assigned male sex at birth and who identify as women) who have sex with men meet to establish contact with clients. Participants in the cohort study were individuals who were assigned male sex at birth and who reported anal sex with another man in the 3 months before enrolment.

For the present study, we included a convenience sample of all participants who ever started PrEP between 19 June 2017 and 30 June 2019.

### Procedures

Cohort procedures have been described elsewhere [12, 13]. In brief, enrolment and monthly follow‐up visits included a face‐to‐face interview using a risk behaviour questionnaire, medical history, physical examination and HIV‐1 counselling and testing using rapid antibody tests. If symptoms of acute HIV‐1 (fever, fatigue, diarrhoea, body pains, sore throat or genital ulcers) or risk criteria [any receptive anal intercourse (RAI), group sex or age (18–29 years)] were met, Xpert^®^ HIV‐1 RNA Qual, Cepheid testing was performed. All participants were provided with syndromic treatment for symptoms suggestive of STIs, received care for minor illnesses as indicated and were vaccinated against hepatitis B virus.

In addition to the face‐to‐face interview, participants completed an inclusion and yearly audio computer‐assisted self‐interview (ACASI) in English or Swahili for depressive symptoms [Patient Health Questionnaire 9 (PHQ‐9)], alcohol use [Alcohol Use Disorder Identification Test (AUDIT)], use of substances other than alcohol and tobacco [Drug Abuse Screening Test 10 (DAST‐10)], sexual stigma (abridged China MSM Stigma Scale), recent trauma (USAID HPI MSM Trauma Screening Tool), gender identity and recent travelling history [14].

Beginning in 19 June 2017, eligible participants were offered free oral daily PrEP (tenofovir‐disoproxil‐fumarate combined with emtricitabine) during their scheduled monthly visits when Kenyan Ministry of Health (MoH) criteria were met for PrEP initiation or cohort‐derived HIV‐1 risk score criteria were met [i.e. a tool developed to guide PrEP initiation among participants, which was based on independent predictors of HIV‐1 infection, including younger age (18–24 years), having only male sex partners, RAI, any unprotected sex and group sex] [2, 15]. Participants were counselled on benefits and risks of PrEP and the importance of adherence, and educated about recognizing symptoms of acute HIV‐1 infection. Those who were willing to start PrEP were provided with a 30‐day supply of PrEP . During monthly visits, PrEP adherence and adverse effects, HIV‐1 status and syndromic STIs were assessed and PrEP refills were provided.

### Outcomes

#### PrEP use

We collected data on whether participants received a PrEP prescription at their monthly visit using pharmacy records. Participants were defined as being on PrEP if they received a PrEP prescription or off PrEP if they did not receive a PrEP prescription at their visit.

#### LTFU

We collected data on the date of visit at every monthly visit. Participants were defined as LTFU if they did not attend a clinic visit for > 90 days.

#### Sexual behaviour

We asked participants whether they had had any RAI and any insertive anal intercourse (IAI) at each monthly visit. Answers to these questions were used to define any RAI and any IAI, respectively. We also asked questions on whether a condom was used 100% or < 100% of the time when engaging in RAI or IAI during the past 3 months. Answers to these questions were used to define a single outcome on condom use, i.e. < 100% condom use during anal sex during the past 3 months.

### Covariates

These were age (18–24, 25+ years), marital status, education, religion, employment status, earnings per month, median asset score, years lived within the region and follow‐up time in the cohort prior to June 2017 (0–6 months, > 6 months). We also obtained the following information at each study visit: sexual behaviour in the past week (no sexual activity, 100% condom use, < 100% condom use for any reported sex acts), number of sex partners in the past month, sex of sex partners in past 3 months (men and women or men only), anal sex practice in the past 3 months (none, insertive, receptive, versatile), transactional sex (receiving or paying for sex) in the past 3 months (yes/no) and area of residence [north coast (closest to KEMRI clinic) or other areas].

From ACASI questionnaires collected yearly, we defined moderate to severe depressive symptoms in the past 2 weeks as having a PHQ‐9 score of 10–27, alcohol use disorder in the past year as having an AUDIT score ≥ 8, and substance use disorder in the past year as having a DAST‐10 score ≥ 1. We measured sexual stigma using a continuous score in the range 0–33. We defined recent trauma as having experienced any mistreatment or violence (score ≥ 1) in the past year. We also obtained information on travel in the past 3 months (yes/no) and gender identity, defined as male, TGW or other.

### Statistical analysis

In analysis, baseline was defined as the visit at which PrEP was initiated. Individual follow‐up started at baseline and continued until the last visit before 30 June 2019, being LTFU or the last HIV‐1‐negative visit (for those with HIV‐1 seroconversion), whichever occurred first. We analysed follow‐up in monthly time intervals. For covariates whose data were missing (e.g. due to questionnaires not being administered at a given time point, participant not returning for their visit, etc.), we assumed that their values did not change until the next assessment (i.e. last observations were carried forward).

We defined three states that participants could occupy during follow‐up: (i) on PrEP; (ii) off PrEP; and (iii) LTFU. The ‘on PrEP’ state included participants who were continuing PrEP use or those who were restarting after having stopped it. The ‘off PrEP’ state included participants who were stopping PrEP or those who remained off PrEP. Cumulative probabilities of stopping PrEP for the first time and of time from being either on PrEP or off PrEP to being LTFU were calculated using the Kaplan–Meier method. Then, using a time‐homogeneous, continuous‐time, multi‐state Markov model, transition intensities (TIs) were modelled and expressed as instantaneous rates per year of a transition occurring (i.e. incidence rate), which depend on the probability of occupying a certain state at each study visit. Transitions were defined as moving from being either on PrEP to off PrEP or vice versa. We simultaneously estimated the TI for transitions from on PrEP to off PrEP (with the transition labelled ‘stopping PrEP’) and from off PrEP to on PrEP (with the transition labelled as ‘restarting PrEP’) separately – and from either PrEP state to being LTFU. Loss to follow‐up was modelled as an absorbing state, i.e. participants were considered to have permanently become LTFU once entering this state. Due to the small number of HIV‐1 seroconversions included in the analysis (*n* = 4), HIV‐1 seroconversion state was not considered as an absorbing state, and we censored observation at the last HIV‐1‐negative visit. We estimated the mean and 95% confidence intervals (CIs) of the number of years spent being on PrEP and off PrEP separately by taking the inverse cumulative TI for each state.

Differences in intensities between levels of determinants were modelled in the multistate Markov model as proportional TIs. Univariable and multivariable hazard ratios (HRs) and their 95% CIs were calculated from these models. Due to possible correlation between sexual behaviour and psychosocial determinants, two separate multivariable models were developed for the following characteristics: (i) demographic and sexual behaviour characteristics (model 1); and (ii) demographics, psychosocial and travelling characteristics (model 2). A forward stepwise selection procedure was used to obtain a final model by sequentially adding variables that were associated with at least one transition in univariable analysis (i.e. whose 95% CI of the HR did not include unity). Any variable whose parameter estimate was unstable (i.e. extremely high or low HR and/or wide 95% CI) was removed from the multivariable model.

We calculated the proportion of individuals reporting: (i) any RAI; (ii) any IAI; and (iii) < 100% condom use during anal sex during the past 3 months. We modelled these endpoints over 3‐month time intervals using a logistic regression model with a random intercept to account for participant variability. *p*‐values for statistical significance were set at *p *< 0.05. Data were analysed using Stata (v.15.0; StataCorp LLC, College Station, TX, USA) and the ‘msm’ package [16] in R (v.3.5; R Core Team).

### Ethical considerations

The KEMRI Ethics Review Committee approved the study. All participants provided written informed consent.

## RESULTS

### Participants characteristics

Between June 2017 and June 2019, 179 HIV‐1‐negative registered MSM and TGW participated in the cohort. Thirty‐six participants (20.1%) never started PrEP and nine (5.0%) reported PrEP use from another PrEP‐providing facility; hence these individuals were excluded from the analysis (Figure S1). In total, 134 participants were included in the analysis. At baseline, participants had a median age of 26 years (range 19–44), 61.2% had secondary education or higher, over half (54.5%) were Christians, nearly 60% used condoms < 100% of the time during anal sex in the past 3 months, nearly two‐thirds (67.2%) reported transactional sex in the past 3 months and nearly one in five identified as women or other gender identity (17.8%) (Table 1).

**TABLE 1:** Baseline characteristics of 134 HIV‐1‐negative participants in Kilifi, Kenya, between June 2017 and June 2019

Characteristics	Total (*n* = 134)
	*n* (%)
Age (years) [median (range)]	26 (19–44)
Age group (years)	
18–24	55 (41.0)
25+	79 (59.0)
Education	
Primary/none	52 (38.8)
Secondary/higher	82 (61.2)
Religion	
Christian	73 (54.5)
Muslim	28 (20.9)
Other/none	33 (24.6)
Employment	
None	21 (15.7)
Self	91 (67.9)
Formal	22 (16.4)
Earnings per month (KSh)	
≥ 10 000	29 (21.6)
< 10 000	105 (78.4)
Asset score [median (IQR)]	3 (2–4)
Time lived in area (years)	
≤ 1	33 (24.6)
> 1	101 (75.4)
Area of residence	
North coast	64 (47.8)
Other areas	70 (52.2)
Follow‐up time in cohort prior to June 2017	
0–6 months	57 (42.5)
> 6 months	77 (57.5)
Sexual behaviour, past week	
No sexual activity	52 (38.8)
100% condom use	53 (39.6)
< 100% condom use	29 (21.6)
Number of sex partners, past month [median (IQR)]	4 (2–7)
Sex of sex partner, past 3 months	
Men and women	62 (46.3)
Men only	72 (53.7)
Anal sex practice, past 3 months	
None	1 (0.7)
Insertive	30 (22.4)
Receptive	38 (28.4)
Versatile	65 (48.5)
Condom use for anal sex, past 3 months	
No anal sex	1 (0.7)
100% condom use	53 (39.6)
< 100% condom use	80 (59.7)
Transactional sex^a^	
No	44 (32.8)
Yes	90 (67.2)
Gender identity^b^	
Male	109 (81.3)
Transgender women/other	24 (17.9)
Alcohol use (AUDIT), past year^b^	
No alcohol use disorder (0–7)	85 (63.4)
Alcohol use disorder (8–40)	48 (35.8)
Depressive symptoms (PHQ‐9), past 2 weeks^b^	
Minimal to mild (0–9)	86 (64.2)
Moderate to severe (10–27)	47 (35.1)
Substance use disorder (DAST−10), past year, (≥ 1)^b^	107 (79.9)
Sexual stigma score (0–33) [median (IQR)]^c^	6 (3–13)
Recent trauma, past year (any)^b^	83 (61.9)
Travelling, past 3 months^b^	74 (55.2)

Abbreviations: AUDIT, alcohol use disorder identification; DAST‐10, Drug Abuse Screening Test 10; KSh, Kenyan shillings; PHQ‐9, Patient Health Questionnaire 9; TGW, transgender women.

^a^ Receiving or paying for sex with cash, living expenses, or goods, past 3 months.

^b^ One missing.

^c^ Four missing.

### Rates of stopping and restarting PrEP

**FIGURE 1:**
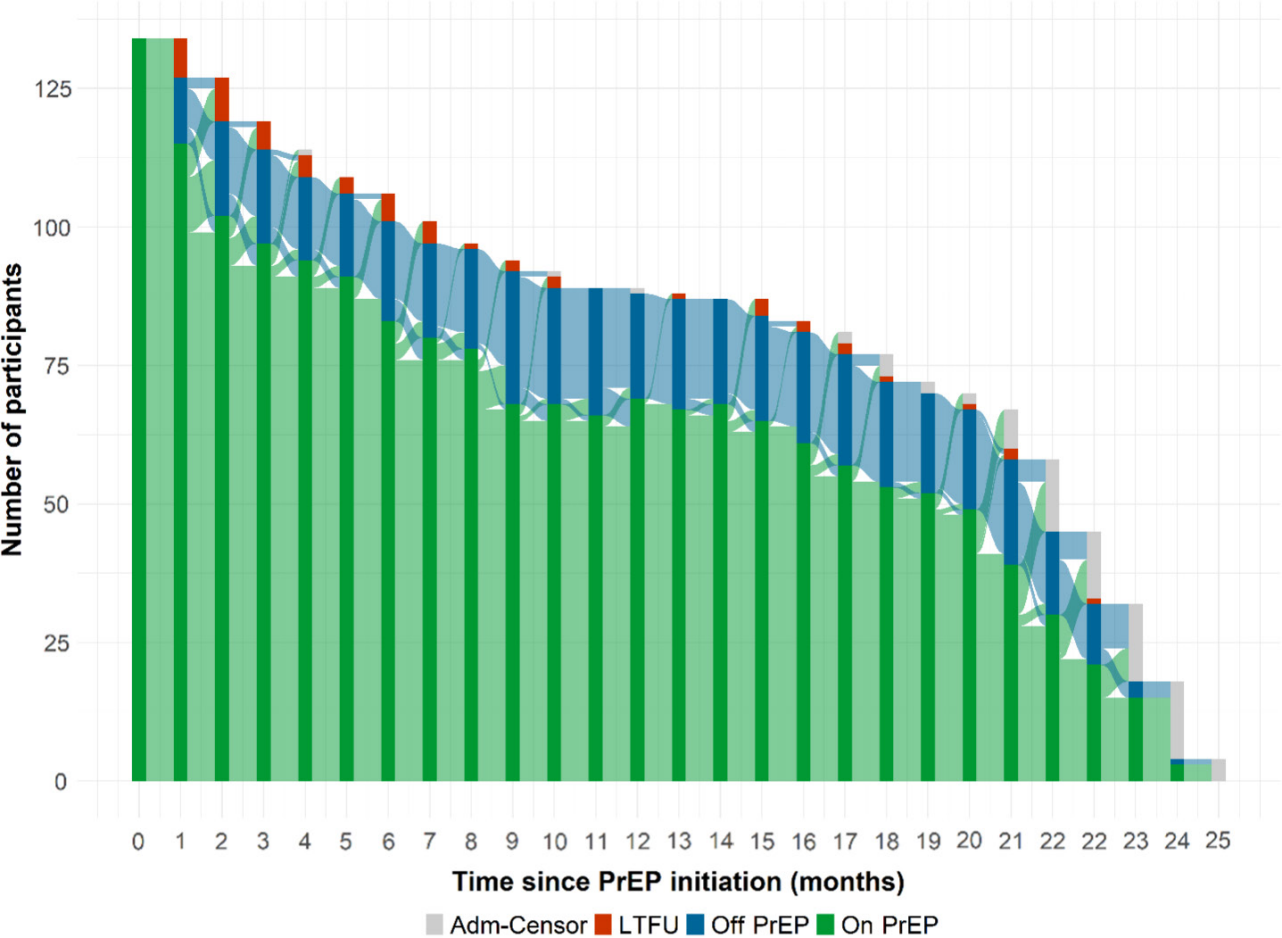
Transitions between pre‐exposure prophylaxis (PrEP) states, and from either pre‐exposure prophylaxis state to being lost to follow‐up (LTFU) among 134 HIV‐1‐negative participants who started PrEP in Kilifi, Kenya, between June 2017 and June 2019. Adm‐Censor, administrative censoring

**FIGURE 2:**
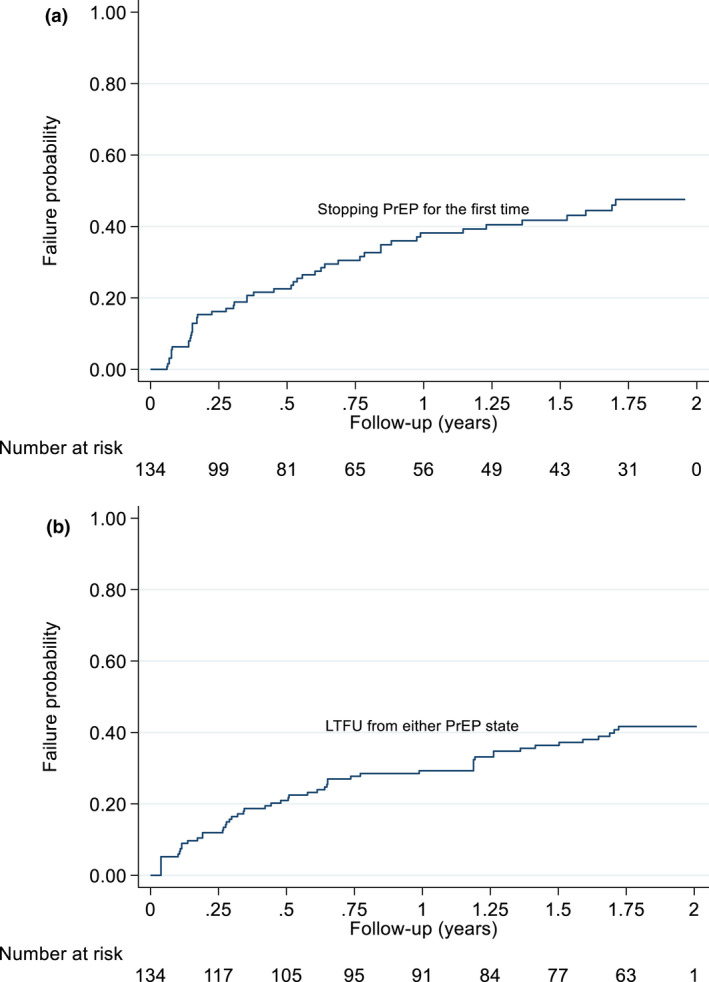
(a, b) Kaplan–Meier failure curve for stopping pre‐exposure prophylaxis (PrEP) for the first time (a) and being lost to follow‐up (LTFU) from either PrEP state (on or off PrEP) (b) among 134 HIV‐1‐negative participants who started PrEP in Kilifi, Kenya between June 2017 and June 2019

Figure 1 describes the distribution of PrEP and LTFU states at each visit and the transitions of states between study visits. A total of 134 participants started PrEP with median initiation month of July 2017 [interquartile range (IQR): June 2017 to August 2017]. Individuals contributed 173.4 person‐years (PY) of follow‐up (median = 20.3 months, IQR: 7.7–22.1 months). Overall, 49 participants stopped PrEP, with the time to the first stop occurring within a median of 129 days (IQR: 55–286) (2‐year cumulative probability = 47.6%, 95% CI: 38.0–58.2%; Figure 2a). Of these, 25 restarted PrEP within a median of 56 days (IQR: 32–84) after stopping for the first time.

**FIGURE 3:**
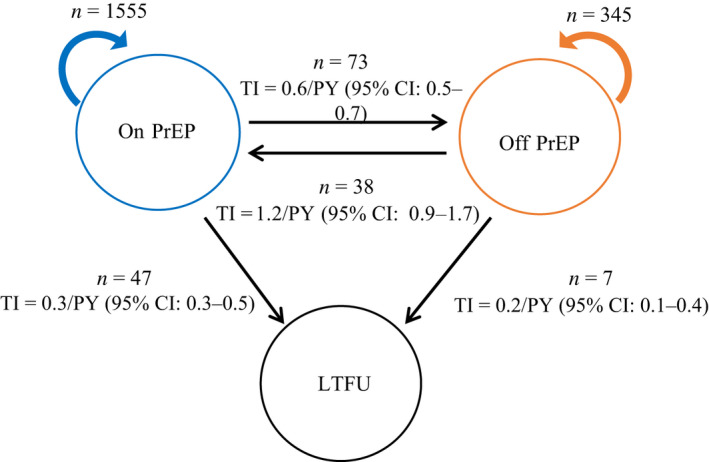
Transitions between being off pre‐exposure prophylaxis (PrEP) and on PrEP and from each PrEP state to being lost to follow‐up (LTFU) among 134 HIV‐1‐negative participants who started PrEP in Kilifi, Kenya between June 2017 and June 2019. *n*, number of follow‐up segments between two visits. Black arrows depict transitions from one state to another occurring between follow‐up visits. Non‐black arrows depict individuals remaining in the same state between follow‐up visits. CI, confidence interval; TI, transition intensity; PY, person‐years

Overall, the 49 participants stopped PrEP 73 times, resulting in a TI of 0.6/PY (95% CI: 0.5–0.7) and restarted PrEP 38 times, resulting in a TI of 1.2/PY (95% CI: 0.9–1.7; Figure 3). Participants spent means of 1.0 (95% CI: 0.9–1.3) and 0.7 (95% CI: 0.5–0.9) years in on PrEP and off PrEP states, respectively, before moving to either state.

In multivariable model 1, restricted to demographics and risk behaviour characteristics, stopping PrEP was related to condom use for anal sex in the past 3 months, and restarting PrEP was associated with non‐Christian or non‐Muslim religion. In multivariable model 2, restricted to demographics, psychosocial and travelling characteristics, stopping PrEP was related to substance use disorder and travelling, and restarting PrEP was related to travelling (Table 2).

**TABLE 2:** Factors associated with stopping pre‐exposure prophylaxis (PrEP) (on PrEP to off PrEP) and restarting PrEP (off PrEP to on PrEP) among 134 HIV‐1‐negative participants who started PrEP in Kilifi, Kenya, between June 2017 and June 2019

Characteristics	Univariable HR		Multivariable HR (model 1^b^)		Multivariable HR (model 2^b^)	
	Off PrEP → on PrEP	On PrEP → off PrEP	Off PrEP → on PrEP	On PrEP → off PrEP	Off PrEP → on PrEP	On PrEP → off PrEP
	HR (95% CI)	HR (95% CI)	aHR (95% CI)	aHR (95% CI)	aHR (95% CI)	aHR (95% CI)
**Demographic characteristics**						
Age group (years)						
18–24	0.7 (0.3–1.9)	0.5 (0.3–1.0)				
25+	Reference	Reference	NI	NI	NI	NI
Education						
Primary/none	Reference	Reference	Reference	Reference	Reference	Reference
Secondary/higher	1.6 (0.8–3.2)	1.0 (0.6–1.5)	1.6 (0.8–3.3)	0.9 (0.6–1.5)	1.6 (0.8–3.4)	0.9 (0.6–1.5)
Religion						
Christian	Reference	Reference	Reference	Reference	Reference	Reference
Muslim	1.9 (0.9–4.0)	1.5 (0.9–2.6)	1.8 (0.8–3.9)	1.3 (0.8–2.4)	1.4 (0.6–3.2)	1.4 (0.8–2.5)
Other/none	**3.5 (1.6–7.7)**	0.7 (0.4–1.4)	**3.6 (1.6–8.3)**	0.7 (0.3–1.3)	1.9 (0.8–4.8)	0.5 (0.3–1.1)
Employment						
None	0.7 (0.2–1.9)	0.7 (0.4–1.4)				
Self	Reference	Reference				
Formal	0.5 (0.1–2.3)	0.5 (0.2–1.0)				
Earnings per month (KSh)						
≥ 10 000	Reference	Reference				
< 10 000	0.6 (0.3–1.2)	1.0 (0.6–1.8)				
Median asset score (IQR)	1.0 (0.8–1.3)	0.9 (0.8–1.1)				
Time lived in area (years)						
≤ 1	0.3 (0.1–1.0)	0.7 (0.3–1.3)	0.4 (0.1–1.5)	0.7 (0.4–1.5)	0.3 (0.1–1.2)	0.6 (0.3–1.2)
> 1	Reference	Reference	Reference	Reference	Reference	Reference
Area of residence						
North coast	Reference	Reference	Reference	Reference	Reference	Reference
Other areas	0.8 (0.4–1.5)	1.1 (0.7–1.7)	1.1 (0.5–2.0)	1.2 (0.7–1.9)	0.8 (0.4–1.6)	1.0 (0.6–1.7)
Follow‐up time in cohort prior to June 2017						
0–6 months	0.3 (0.1–0.8)	0.6 (0.3–1.0)				
> 6 months	Reference	Reference	NI	NI	NI	NI
**Risk behaviour characteristics**						
Sexual behaviour, past week						
No activity	Reference	Reference				
100% condom use	0.8 (0.4–1.7)	0.7 (0.4–1.2)				
< 100% condom use	1.0 (0.4–2.2)	0.9 (0.5–1.6)				
Number of sex partners, past month	1.1 (0.8–1.5)	1.0 (0.7–1.3)				
Sex of sex partner, past 3 months						
Men and women	Reference	Reference	Reference	Reference		
Men only	1.0 (0.5–1.8)	0.6 (0.4–1.0)	1.1 (0.5–2.1)	0.7 (0.4–1.2)		
Anal sex practice, past 3 months						
None	1.8 (0.5–6.4)	1.3 (0.5–3.5)	NI	NI		
Insertive	Reference	Reference				
Receptive	0.7 (0.3–1.9)	0.6 (0.3–1.2)				
Versatile	1.1 (0.5–2.3)	0.6 (0.4–1.1)				
Condom use for anal sex, past 3 months						
No anal sex	2.2 (0.6–7.8)	**3.3 (1.3–8.2)**	1.6 (0.4–6.3)	**3.1 (1.2–8.2)**		
100% condom use	Reference	Reference	Reference	Reference		
< 100% condom use	1.4 (0.7–2.8)	**1.7 (1.0–2.8)**	1.2 (0.6–2.4)	**1.7 (1.0–2.9)**		
Transactional sex^a^						
No	Reference	Reference				
Yes	1.0 (0.5–1.9)	0.9 (0.6–1.4)				
**Psychosocial characteristics**						
Gender identity						
Male	Reference	Reference				
TGW/other	0.5 (0.2–1.5)	0.6 (0.3–1.1)				
Depressive symptoms (PHQ‐9), past 2 weeks						
Minimal to mild (0–9)	Reference	Reference				
Moderate to severe (10–27)	0.5 (0.2–1.1)	0.8 (0.5–1.3)				
Alcohol use (AUDIT), past year						
No alcohol use disorder (0–7)	Reference	Reference			Reference	Reference
Alcohol use disorder (8–40)	**2.0 (1.1–3.9)**	1.2 (0.7–1.9)			1.4 (0.6–2.9)	0.9 (0.5–1.5)
Substance use disorder (DAST‐10), past year (≥ 1)	2.0 (0.8–5.1)	**1.8 (1.0–3.4)**			1.0 (0.3–3.2)	**2.2 (1.1–4.2)**
Sexual stigma score (0–33)	1.0 (0.7–1.7)	1.3 (0.9–1.7)				
Recent trauma, past year (any)	**2.7 (1.4–5.5)**	1.5 (0.9–2.4)			NI	NI
Travelling, past 3 months	**3.0 (1.5–6.1)**	**2.0 (1.3–3.3)**			**3.3 (1.4–7.5)**	**2.3 (1.4–3.8)**

Bolded values indicate statistically significant difference (*p* < 0.05).

Abbreviations: aHR, adjusted hazard ratio; AUDIT, alcohol use disorder identification; CI, confidence interval; DAST‐10, Drug Abuse Screening Test 10; HR, hazard ratio; KSh, Kenyan shillings; NI, not included in multivariable analysis due to unstable parameter estimates; PHQ‐9, Patient Health Questionnaire 9; TGW, transgender women.

^a^ Receiving or paying for sex with cash, living expenses or goods over past 3 months.

^b^ Model 1, demographic and sexual behaviour characteristics; model 2, demographic, psychosocial and travel characteristics.

### Becoming LTFU from either PrEP state

A total of 54 (40.3%) participants were LTFU (2‐year cumulative probability = 41.7%, 95% CI: 33.7–50.8%) within a median of 5.5 months (IQR: 2.1–14.3 months; Figure 2b). Of these, 47 participants transitioned to being LTFU from a previous on PrEP state and seven from a previous off PrEP state. The TI for being LTFU while on PrEP was 0.3/PY (95% CI: 0.3–0.5) and while off PrEP was 0.2/PY (95% CI: 0.1–0.4). Rates of being LTFU did not differ by PrEP state (Figure 3).

In multivariable model 1, restricted to demographics and risk behaviour characteristics, being LTFU while on PrEP was associated with secondary education or higher, living in the area for ≤ 1 year and residence outside the immediate clinic catchment area. In multivariable model 2, restricted to demographics, psychosocial and travelling characteristics, being LTFU while on PrEP was associated with living in the area for ≤ 1 year, residence outside the immediate clinic catchment area and alcohol use disorder. No variables were significantly associated with being LTFU while off PrEP (Table 3).

**TABLE 3:** Factors associated with being lost to follow‐up (LTFU) from either pre‐exposure prophylaxis (PrEP) state among 134 HIV‐1‐negative participants who started PrEP, in Kilifi, Kenya, between June 2017 and June 2019

Characteristics	Univariable HR		Multivariable HR (model 1^b^)		Multivariable HR (model 2^b^)	
	On PrEP → LTFU	Off PrEP → LTFU	On PrEP → LTFU	Off PrEP → LTFU	On PrEP → LTFU	Off PrEP → LTFU
	HR (95% CI)	HR (95% CI)	aHR (95% CI)	aHR (95% CI)	aHR (95% CI)	aHR (95% CI)
**Demographic characteristics**						
Age group (years)						
18–24	1.6 (0.9–2.8)	**6.7 (1.4–32.3)**				
25+	Reference	Reference	NI	NI	NI	NI
Education						
Primary/none	Reference	Reference	Reference	Reference	Reference	Reference
Secondary/higher	**2.3 (1.1–4.5)**	0.7 (0.1–3.2)	**2.1 (1.0–4.1)**	0.1 (0.0–27.4)	1.7 (0.9–3.5)	0.9 (0.1–6.3)
Religion						
Christian	Reference	Reference	Reference	Reference	Reference	Reference
Muslim	0.9 (0.4–2.0)	0.4 (0.0–4.6)	1.2 (0.6–2.5)	^c^	1.3 (0.6–2.9)	0.4 (0.0–5.0)
Other/none	1.0 (0.5–1.9)	1.0 (0.1–10.1)	1.3 (0.6–2.5)	0.3 (0.0–9.1)	1.2 (0.6–2.3)	1.0 (0.1–10.3)
Employment						
None	0.8 (0.4–1.9)	2.3 (0.2–22.0)				
Self	Reference	Reference				
Formal	1.0 (0.5–2.2)	2.8 (0.5–16.4)				
Earnings per month (KSh)						
≥ 10 000	Reference	Reference				
< 10 000	0.7 (0.4–1.3)	1.4 (0.1–13.9)				
Median asset score (IQR)	1.1 (0.9–1.4)	1.0 (0.6–1.5)				
Time lived in area (years)						
≤ 1	**2.7 (1.5–4.9)**	1.5 (0.3–8.2)	**2.1 (1.1–3.9)**	1.9 (0.2–23.2)	**2.3 (1.2–4.4)**	1.2 (0.2–7.3)
> 1	Reference	Reference	Reference	Reference	Reference	Reference
Area of residence						
North coast	Reference	Reference	Reference	Reference	Reference	Reference
Other areas	**3.3 (1.7–6.3)**	1.2 (0.3–5.8)	**2.8 (1.4–5.6)**	0.1 (0.0–10.6)	**2.7 (1.4–5.4)**	1.3 (0.2–8.1)
Follow‐up time in cohort prior to June 2017						
0–6 months	**4.5 (2.4–8.3)**	**6.3 (1.1–35.1)**	NI	NI	NI	NI
> 6 months	Reference	Reference				
**Risk behaviour characteristics**						
Sexual behaviour, past week						
No activity	Reference	Reference				
100% condom use	1.0 (0.5–1.9)	0.5 (0.1–3.0)				
< 100% condom use	0.5 (0.2–1.2)	0.8 (0.1–5.3)				
Number of sex partners, past month	1.1 (0.8–1.5)	1.2 (0.5–2.7)				
Sex of sex partner, past 3 months						
Men and women	Reference	Reference	Reference	Reference		
Men only	**1.9 (1.0–3.6)**	1.6 (0.3–7.4)	1.5 (0.8–2.9)	1.1 (0.1–12.5)		
Anal sex practice, past 3 months						
None	1.5 (0.3–7.7)	6.9 (0.4 to > 100.0)				
Insertive	Reference	Reference	NI	NI		
Receptive	**2.6 (1.0–6.5)**	1.4 (0.1–27.1)				
Versatile	1.5 (0.6–3.7)	3.2 (0.3–31.9)				
Condom use for anal sex, past 3 months						
No anal sex	0.5 (0.1–4.2)	3.2 (0.3–32.6)	1.0 (0.1–8.0)	10.8 (0.4‐> 100.0)		
100% condom use	Reference	Reference	Reference	Reference		
< 100% condom use	0.9 (0.5–1.6)	0.9 (0.2–4.7)	0.9 (0.5–1.6)	0.9 (0.1–6.5)		
Transactional sex^a^						
No	Reference	Reference				
Yes	1.6 (0.8–3.1)	1.7 (0.3–9.2)				
**Psychosocial characteristics**						
Gender identity						
Male	Reference	Reference				
TGW/other	0.9 (0.5–1.9)	^c^				
Depressive symptoms (PHQ‐9), past 2 weeks						
Minimal to mild (0–9)	Reference	Reference				
Moderate to severe (10–27)	1.1 (0.6–2.0)	1.5 (0.3–7.0)				
Alcohol use (AUDIT), past year						
No alcohol use disorder (0–7)	Reference	Reference			Reference	Reference
Alcohol use disorder (8–40)	**2.7 (1.5–4.8)**	0.3 (0.0–3.9)			**2.3 (1.2–4.6)**	0.2 (0.0–2.4)
Substance use disorder (DAST‐10), past year (≥ 1)	1.9 (0.9–4.0)	1.7 (0.2–15.0)			1.3 (0.5–3.0)	2.8 (0.3–32.2)
Sexual stigma score (0–33)	1.2 (0.9–1.8)	0.4 (0.1–1.1)				
Recent trauma, past year (any)	1.3 (0.7–2.4)	7.4 (0.8–72.5)			NI	NI
Travelling, past 3 months	1.1 (0.6–2.0)	0.8 (0.2–4.0)			0.8 (0.4–1.5)	0.7 (0.1–3.9)

Bolded values indicate statistically significant difference (*p* < 0.05).

Abbreviations: aHR, adjusted hazard ratio; AUDIT, Alcohol Use Disorder Identification; CI, confidence interval; DAST‐10, Drug Abuse Screening Test 10; HR, hazard ratio; KSh, Kenyan shillings; NI, not included in multivariable analysis due to unstable parameter estimates; PHQ‐9, Patient Health Questionnaire‐9; TGW, transgender women.

^a^ Receiving or paying for sex with cash, living expenses or goods over the past 3 months.

^b^ Model 1, demographic and sexual behaviour characteristics; model 2, demographic, psychosocial and travel characteristics.

^c^ < 0.0 (< 0.0 to > 100.0).

### Sexual risk behaviour during follow‐up by PrEP use

**FIGURE 4:**
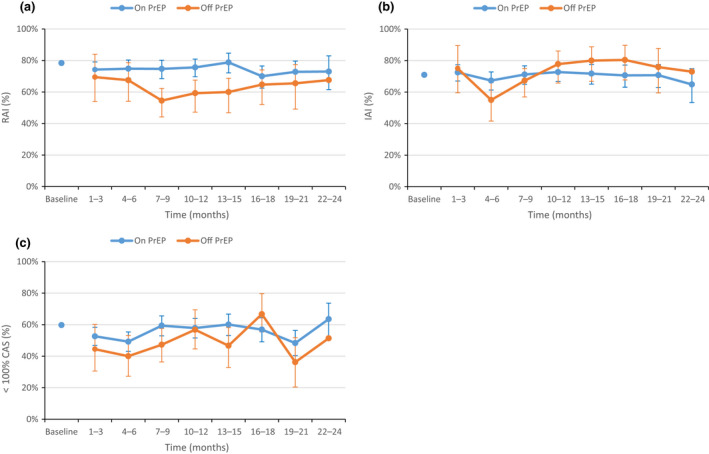
(a–c) Proportion of any receptive anal intercourse (RAI) (a), insertive anal intercourse (IAI) (b), and < 100% condom use for anal sex (CAS) (c) in the past 3 months among 134 HIV‐1‐negative participants in Kilifi, Kenya, between June 2017 and June 2019. PrEP, pre‐exposure prophylaxis.

Of 134 participants who ever started PrEP, 105 (78.4%) reported any RAI, 95 (70.9%) reported any IAI, and 80 (59.7%) reported < 100% condom use during anal sex at baseline. Overall, no statistically significant difference was observed in the proportion of individuals with any RAI [odds ratios (OR) = 1.21 (0.70−2.07), *p* = 0.49] during on PrEP visits compared with off PrEP visits (Figure 4a). The proportion of participants with any IAI was higher during off PrEP visits [OR = 1.81 (1.11−2.30), *p* = 0.02] than during on PrEP visits (Figure 4b). With respect to the proportion of participants reporting < 100% condom use during anal sex, this proportion was lower during off PrEP visits than during on PrEP visits [OR = 0.63 (0.45−0.87), *p *< 0.01] (Figure 4c).

## DISCUSSION

In a cohort of PrEP‐taking MSM and TGW followed up for approximately 2 years in coastal Kenya, almost half stopped PrEP and, of these, half re‐started. We found that stopping PrEP was related to condom use for anal sex and substance use disorder, and restarting PrEP was associated with non‐Christian or non‐Muslim religion. Travelling was related to frequently transitioning between being on and off PrEP. While the rate of being LTFU did not differ by PrEP state, a substantial proportion of participants were LTFU (41.7%), consistent with previous reports from this cohort. Being LTFU while on PrEP was associated with having a secondary education or higher, living in the study area for ≤ 1 year, residence outside the immediate clinic catchment area and alcohol use disorder. We also found that participants reported higher proportions of IAI and lower proportions of inconsistent condom use for anal sex during off PrEP visits.

We found that stopping PrEP was related to substance use disorder. Among African MSM, substance use disorder is prevalent, and men mostly use drugs in combination with alcohol during sexual encounters, often leading to delays in taking pills or missed PrEP doses [17]. Targeted and individualized counselling and efforts to reduce substance use among MSM are needed, including integrating screening, counselling and referral for mental health challenges and harm reduction programmes for substance use in PrEP‐providing facilities [14].

Stopping and restarting PrEP was related to travelling in this study. In a published study of PrEP use among MSM and female sex workers (FSW) followed at the same research clinic as the current analysis, mobility was also linked to missed PrEP doses and poor adherence [17]. A similar effect of ART interruption among HIV‐1‐positive FSW due to mobility was reported across different settings [18]. Additionally, participants may restart PrEP after returning from travel (possibly because of heightened risk awareness) [19]. Our findings suggest that electronic and other media technologies for linkage of MSM to the nearest HIV‐1 prevention and care services are needed [20].

Restarting PrEP was more frequent among participants with non‐Christian or non‐Muslim religious affiliation in this study. Why men not affiliated to any Christian or Muslim religion were more likely to restart PrEP is unclear. In a literature review of 29 studies in men and women, a positive association of religion, faith and spirituality was found with HIV prevention uptake, such as condom use and HIV testing [21], which is contrary to our findings. Nevertheless, religious leaders who are sensitized to sexual minorities may play a role in their HIV‐1 prevention and care [22]. It should be noted that condom use is very different from PrEP as an HIV prevention tool and perhaps the populations interested in PrEP are different from those in these previous studies. Further qualitative assessments to understand sociocultural and behavioural factors that influence PrEP uptake, continuation and discontinuation are needed in this setting.

In this study, we found that participants who reported inconsistent condom use for anal sex and those who did not report anal sex were more likely to stop PrEP during follow‐up. While participants who did not report anal sex may discontinue PrEP due to changes in sexual risk behaviour, it is concerning that participants who reported inconsistent condom use during anal sex were more likely to stop PrEP. However, this finding must be considered in light of the higher proportions of individuals reporting IAI and lower proportions of individuals reporting inconsistent condom use for anal sex during visits while off PrEP compared with visits while on PrEP, which may indicate lower risk behaviours among men off PrEP. These findings suggest that Kenyan MSM who are not at risk of HIV‐1 on a daily basis and are able to plan for sex may prefer a more flexible and convenient PrEP‐dosing strategy, especially if they encounter challenges in taking daily PrEP. In this instance, event‐driven PrEP might align more closely with risk behaviours and individual preferences [23]. Of note, event‐driven PrEP has not been promoted in Kenya and is not included in the current Kenyan PrEP guidelines [2].

We found that being LTFU was most frequent among individuals who were on PrEP, although we did not find significant differences in LTFU rates according to PrEP state. Determinants of becoming LTFU while on PrEP were: alcohol use disorder, residing further from the research clinic and having a secondary level of education or higher, consistent with recent findings in this study [24]. In addition, we found that being LTFU while on PrEP was more likely among men who resided in the area for 1 year or less. Among MSM followed up in an HIV‐1 risk reduction program in Kisumu, Kenya, missing two follow‐up visits was more common among men who resided in the area for less than a year [25]. In the present study, we did not identify predictors for being LTFU while off PrEP, possibly owing to the lack of statistical power in identifying determinants from the small number of participants. However, our results suggest that linking MSM who have recently moved into an area to local MSM‐friendly services for social support and PrEP care services, screening for mental health and strengthened engagement may improve both retention in HIV‐1 prevention services and adherence to PrEP [24, 25, 26].

Our study has several limitations. First, PrEP use was based on pharmacy refill data. Participants who received a PrEP refill but did not take PrEP would have been misclassified as PrEP users. In addition, we did not collect data on PrEP adherence, and therefore we could not use this information to categorize participants as being on PrEP. Second, we could not determine if participants who were LTFU while on PrEP re‐engaged with PrEP care at another PrEP‐providing facility. However, due to the accessibility of LGBTI+‐friendly PrEP programmes in major towns in Kenya, including Nairobi and Kisumu, it is probable that some participants accessed PrEP services elsewhere. Third, we did not obtain qualitative assessment of reasons for stopping and restarting PrEP. Fourth, the face‐to‐face risk behavioural interview may have been subject to social desirability bias. However, psychosocial and other characteristics collected via ACASI resulted in identification of important determinants (i.e. substance use disorder and alcohol use disorder) associated with switching between PrEP states and being LTFU. Fifth, we did not test systematically for STIs using nucleic acid amplification testing‐based screening and our behaviour assessment was based on self‐report. Sixth, the small number of participants at certain levels of determinants and the few transitions between certain states may have limited the power to detect some statistically significant determinants. Finally, MSM participants followed in this study had access to tailored HIV‐1 prevention services, and therefore may not be representative of all MSM in Kenya.

## CONCLUSIONS

In this coastal Kenya cohort, switching between being on and off PrEP was common, and participants at higher risk due to condomless anal sex and substance use disorder were more likely to stop PrEP. However, fewer participants reported high‐risk behaviours while off PrEP. Being LTFU was common in this population and was related to alcohol use and structural barriers, such as residing further from the clinic area and years lived within the area. Screening for mental health, linking MSM and TGW to local MSM and transgender‐friendly services for social support and strengthened engagement may improve retention in HIV‐1 prevention services. In addition, alternative regimens, including event‐driven PrEP or long‐acting injectable PrEP for MSM and TGW with PrEP‐taking challenges, may improve PrEP adherence.

## CONFLICTS OF INTEREST

MP reports grants from Gilead sciences, Roche, AbbVie and MSD during the conduct of the study.

## AUTHOR CONTRIBUTIONS

ES and SMG designed the research study. AT, KM and JM collected the data. TO and EW managed the data, and EW and AB analysed the data. EW wrote the original draft of the manuscript. AB, EvdE, SMG, MP and ES critically reviewed and edited the manuscript. All authors read and provided feedback on manuscript drafts and approved the final manuscript.

## Supporting information

Fig S1
