# Supplementary material for: Stopping and restarting PrEP and loss to follow‐up among PrEP‐taking men who have sex with men and transgender women at risk of HIV‐1 participating in a prospective cohort study in Kenya
Source: HIV Med. 2022 Jan 27;23(7):750–63. doi: 10.1111/hiv.13237 (PMC9276557; doi:10.1111/hiv.13237)
Supplement: Supplementary file 1 — Fig S1 [file HIV-23-750-s001.docx]

**Supplementary Figure 1. Number of MSM and transgender women included in analysis, Kilifi, Kenya, June 2017-June 2019.**
